# Unintended Interruption of Caplacizumab Therapy Can Compromise Outcomes in Immune-Mediated Thrombotic Thrombocytopenic Purpura: Lessons from Two Clinical Cases

**DOI:** 10.3390/jcm15176601

**Published:** 2026-08-26

**Authors:** Fedai Özcan, Fahri Kiziler, Paul Brinkkoetter, Lucas Kühne, Paul Knöbl, Alexandra Brinkhoff

**Affiliations:** 1 Department of Nephrology, Klinikum Dortmund, Faculty of Medicine, University of Witten-Herdecke, 44137 Dortmund, Germany; 2 Department II of Internal Medicine, University Hospital Cologne, Faculty of Medicine, University of Cologne, 50937 Cologne, Germany; paul.brinkkoetter@uk-koeln.de (P.B.);; 3 Center for Molecular Medicine Cologne, University Hospital Cologne, Faculty of Medicine, University of Cologne, 50931 Cologne, Germany; 4 Division of Hematology, Department of Medicine 1, Medical University of Vienna, 1090 Vienna, Austria; paul.knoebl@meduniwien.ac.at

**Keywords:** caplacizumab, thrombotic thrombocytopenic purpura, treatment interruption, ADAMTS13 activity

## Abstract

**Background:** Caplacizumab is a cornerstone in the management of immune-mediated thrombotic thrombocytopenic purpura (iTTP), in combination with plasma exchange (PEX) and immunosuppression. Pivotal trials and real-world data have demonstrated faster platelet count recovery and improved remission rates. Nevertheless, a subset of patients still experiences exacerbations or delayed response. Observational evidence suggests that unintended treatment interruptions may contribute to these unfavorable outcomes. **Methods:** We report on two patients with iTTP treated with caplacizumab and immunosuppression; PEX was used in Case 1 and as escalation therapy in Case 2. Laboratory parameters including platelet count, lactate dehydrogenase (LDH), ADAMTS13 activity, and anti-ADAMTS13 autoantibodies were monitored. **Results**: In the first case, a 29-year-old female experienced an exacerbation and ischemic complications after two individual caplacizumab doses were unintentionally missed on days 12 and 14. Subsequent continuous administration led to stabilization and recovery. In the second case, a 42-year-old male presented with hemolytic anemia, thrombocytopenia, and ADAMTS13 activity of 1% and was initially treated with prednisolone and caplacizumab without PEX. Two individual caplacizumab doses were unintentionally missed on days 3 and 5 and were followed by delayed treatment response and microembolic infarcts. Treatment escalation to PEX and rituximab and reinitiation of continuous caplacizumab administration led to clinical stabilization. **Conclusions:** Even brief interruptions of caplacizumab therapy may compromise outcomes in iTTP. Continuous administration and secured availability, together with serial monitoring of ADAMTS13 activity to guide treatment duration and discontinuation, are essential to improve patient care.

## 1. Background

Caplacizumab inhibits the ultra-large von Willebrand factor (UL-VWF)-mediated aggregation of platelets and has become a key component in the management of immune-mediated thrombotic thrombocytopenic purpura (iTTP). Commonly, caplacizumab is added to the recommended therapeutic backbone consisting of plasma exchange (PEX) and glucocorticoid-based immunosuppression [1]. PEX removes pathological UL-VWF multimers and autoantibodies against ADAMTS13 (a disintegrin and metalloproteinase with a thrombospondin type 1 motif member 13) and replenishes ADAMTS13. A combination of PEX and immunosuppression has enabled normalization of platelet counts in approximately 95% of patients and disease remission in 87% [2]. Nevertheless, exacerbations and relapses of iTTP remain common, with reported rates of up to 50% [3]. The addition of caplacizumab has further reduced exacerbation and relapse rates. In the pivotal TITAN and HERCULES trials, caplacizumab led to significantly faster normalization of platelet counts compared with a placebo and a higher proportion of patients achieved complete remission, with exacerbation rates below 10% [4,5]. These results have also been corroborated by real-world evidence [6]. Importantly, clinical improvement and platelet-count normalization do not necessarily indicate control of the underlying autoimmune process. Persistently low ADAMTS13 activity reflects ongoing disease activity, and observational studies have shown that cessation of caplacizumab while ADAMTS13 activity remains severely reduced (e.g., <10%) is associated with an increased risk of exacerbation or relapse [7,8]. Thus, serial ADAMTS13 measurements should complement clinical and conventional laboratory parameters when decisions regarding caplacizumab treatment duration and discontinuation are made. We hypothesize that treatment cessation or interruption of caplacizumab (intended or unintended) is more likely to occur under real-world conditions than in a controlled clinical trial setting and may increase the risk of iTTP exacerbation or relapse. We therefore retrospectively analyzed iTTP cases from our clinic in which exacerbations or complications occurred. The following two cases illustrate clinical scenarios in which unintended interruptions of caplacizumab were associated with disease exacerbation (Case 1) and delayed response and complications (Case 2).

## 2. Materials and Methods

This retrospective two-case analysis included patients treated for a first acute episode of iTTP at Klinikum Dortmund, University of Witten/Herdecke, Dortmund, Germany, in 2022 (Case 1) and 2024 (Case 2). During the acute phase, because of the risk of complications, particularly thromboembolic events, both patients were initially managed in the medical intensive care unit and transferred to the nephrology ward after clinical stabilization. The diagnosis of iTTP was based on thrombocytopenia and microangiopathic hemolytic anemia with schistocytes, together with severe ADAMTS13 deficiency (<10%) and detectable anti-ADAMTS13 autoantibodies. The PLASMIC score was 6 in both patients. Anti-ADAMTS13 autoantibodies were measured using an ELISA-based assay (Technoclone^®^, ADAMTS13 INH Elisa, Vienna. Austria). Data were retrospectively extracted from the medical records and included the clinical course, platelet count, LDH, ADAMTS13 activity, anti-ADAMTS13 autoantibody levels, caplacizumab, PEX, rituximab and prednisolone treatment, as well as relevant imaging findings and complications. Written informed consent for publication of the clinical data and accompanying images was obtained from both patients. According to institutional requirements, formal ethics committee approval was not required for the retrospective description of these individual cases.

## 3. Case Presentation

**Figure 1:**
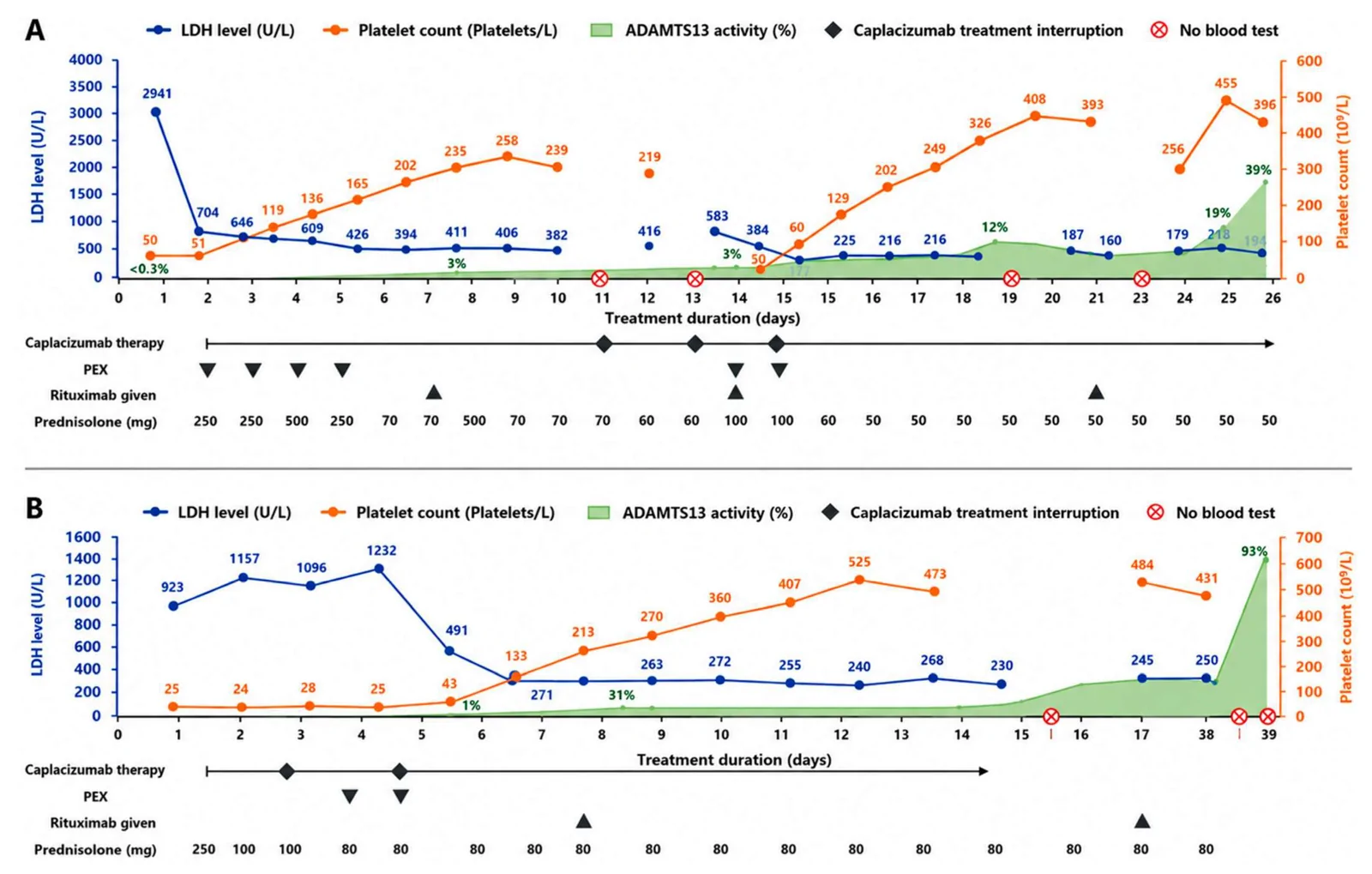
Laboratory markers and treatment course for TTP during caplacizumab treatment in two cases. Changes in platelet count (orange line) and LDH levels (blue line) over the course of caplacizumab treatment in Case 1 (**A**) and Case 2 (**B**). ADAMTS13 activity (%) is represented by green shading. Unintended caplacizumab treatment interruptions are indicated by diamonds, days with plasma exchange (PEX) are indicated by down-pointing triangles and administrations of rituximab are indicated by up-pointing triangles, respectively.

Here we report on two cases of iTTP treated with caplacizumab and immunosuppression; PEX was administered as part of the treatment course as detailed below. Laboratory parameters, including LDH activity, platelet count, ADAMTS13 activity, and anti-ADAMTS13 autoantibody levels, together with an overview of the treatment course, are presented in Figure 1.

Case 1. A 29-year-old female patient presented with impaired consciousness (Glasgow Coma Scale 8) and required intubation and ventilation. During hospitalization, she experienced circulatory arrest necessitating two minutes of resuscitation. The initial platelet count was 15,000/µL. Thrombocytopenia, schistocytes, and hemolytic anemia led to the presumptive diagnosis of TTP, which was rapidly confirmed by an ADAMTS13 activity of <0.3% and elevated anti-ADAMTS13 autoantibodies of 56 U/mL (positive ≥16 U/mL). The patient was treated with PEX for 5 days and prednisolone continuously; caplacizumab was administered uninterrupted for 11 days. Three doses of rituximab were given on days 6, 13, and 20. Under therapy, platelet count increased progressively while LDH levels decreased. On days 12 and 14, one daily dose of caplacizumab was unintentionally missed on each day. These interruptions were associated with fulminant disease exacerbations with a platelet count drop to 20,000/µL and an increase in LDH to 583 U/L, after which additional PEX was performed and the prednisolone dose was temporarily increased. In total, PEX was performed 6 times. Reinitiation of continuous caplacizumab administration on day 15 led to progressive stabilization of platelet counts and recovery of ADAMTS13 activity. Anti-ADAMTS13 autoantibody levels were 55 U/mL, 58 U/mL, 12 U/mL, 10 U/mL, and 9 U/mL on subsequent measurements, while ADAMTS13 activity increased to 39%. Caplacizumab therapy was terminated on day 26 when ADAMTS13 activity was 39% (24 doses in total). On day 29, the patient was discharged in good physical condition without significant neurological abnormalities.

Case 2. A 42-year-old male patient presented with nausea, vomiting, night sweats, and a weight loss of 5 kg over 14 days. The initial platelet count was 24,000/µL. Laboratory findings indicated hemolytic anemia, thrombocytopenia, and fragmentocytes, raising suspicion of TTP, which was confirmed by an ADAMTS13 activity of 1% and anti-ADAMTS13 autoantibodies of 41 U/mL. Treatment was initiated with a three-day prednisolone pulse and caplacizumab, initially without PEX. There was no contraindication to PEX or central venous catheter placement. After discussion with the patient, a deliberate PEX-free treatment strategy with caplacizumab and immunosuppression under close clinical and laboratory monitoring was initially selected, based on published PEX-free treatment approaches in selected patients [9,10]. PEX was planned as escalation therapy in the event of an insufficient response or clinical deterioration. Despite treatment initiation, the platelet count remained critically low. One daily caplacizumab dose was unintentionally missed on day 3 and another on day 5; on day 4, the platelet count dropped to 25,000/µL, while ADAMTS13 activity remained low at 1%. Therapy was therefore escalated with PEX on days 4 and 5. After the two PEX procedures and re-establishment of continuous caplacizumab administration from day 6, platelet counts and ADAMTS13 activity progressively increased while LDH activity decreased. Anti-ADAMTS13 autoantibodies decreased from 41 U/mL at presentation to 1 U/mL on subsequent measurements; ADAMTS13 activity increased to 31% and later to 93%. Two doses of rituximab were given on days 7 and 14. On day 7, elevated troponin levels raised concern regarding possible myocardial involvement. Coronary heart disease was ruled out, but neurological symptoms (facial and right arm paresthesia) prompted magnetic resonance imaging, revealing microembolic infarcts. Caplacizumab was discontinued on day 12 when ADAMTS13 activity was 31% (9 doses in total). Three weeks later, ADAMTS13 activity reached 93%, with no new ischemic events or persistent neurological deficits, confirming a favorable clinical outcome.

## 4. Discussion and Conclusions

We report on two patients with iTTP for whom unintended interruptions of caplacizumab led to disease exacerbation (case 1) or a delayed response and complications (case 2). In a recent 2026 analysis of the German REACT-2020 and Austrian ATMAR cohorts, missing doses of caplacizumab were indeed among the central reasons for either diseases refractoriness or delayed response [11].

In Case 1, caplacizumab interruption occurred after completion of the initial PEX course and transfer from the intensive care unit to the general ward on day 8, after stabilization of the major clinical and laboratory parameters. The subsequent fulminant platelet count decrease required retransfer to the ICU and reinitiation of PEX. Retrospective review of the treatment course suggested that the interruption was most likely related to drug availability on the general ward and communication with the in-house pharmacy; the exact cause could not be determined with certainty. In Case 2, PEX was initially omitted deliberately after discussion with the patient, using a PEX-free caplacizumab-based strategy under close monitoring as previously described in selected patients [9,10]. PEX remained a predefined escalation option and was initiated promptly when the response was insufficient. The absence of concomitant PEX may have increased the clinical relevance of the missed caplacizumab doses, because VWF-mediated platelet adhesion remained dependent on continuous caplacizumab activity while ADAMTS13 activity was still severely reduced. Thus, the unintended interruption of caplacizumab in this setting may have contributed to the delayed response and ischemic complications. Retrospective review showed that the first interruption was related to drug unavailability, whereas the reason for the second interruption was not documented.

It is remarkable that these negative outcomes occurred after only brief treatment interruptions, considering that Kühne et al. reported on a safe switch to alternate-day dosing of caplacizumab, taking into account caplacizumab’s pharmacodynamic properties [12]. However, in both cases, interruptions occurred when ADAMTS13 activity was still very low (<5–10%), indicating that interruptions of caplacizumab in this phase are especially problematic as has been observed before [7,8]. Indeed, a 2023 analysis demonstrated discontinuation of caplacizumab at an ADAMTS13 activity ≤10% was associated with complications in 44% of analyzed patients (*n* = 19/43), most frequently exacerbations, whereas no complications occurred in patients with an ADAMTS13 activity >10% at capalcizumab cessation (*n* = 76) [13]. Patients may therefore be particularly vulnerable to caplacizumab interruptions during the first few days of therapy when immunological activity is high and ADAMTS13 activity still low.

**Figure 2:**
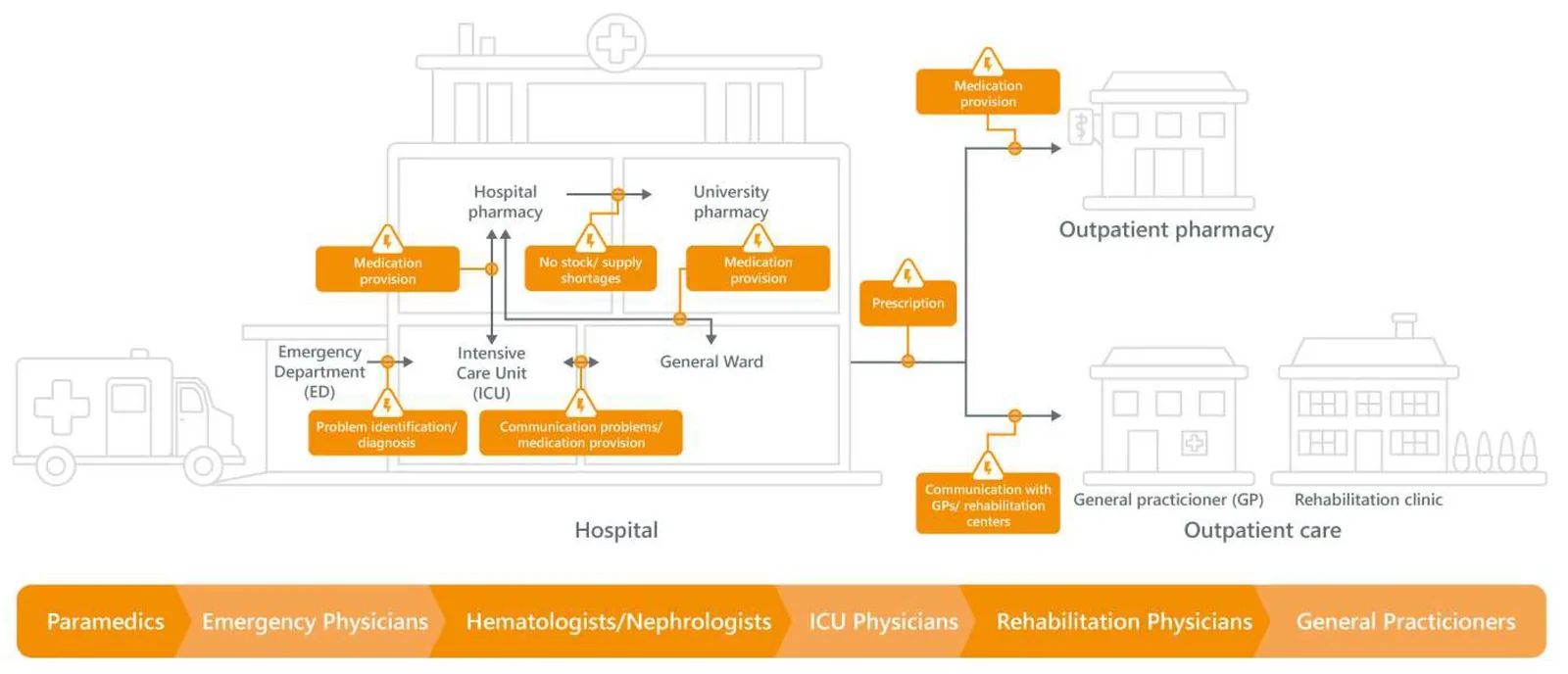
Critical points for uninterrupted caplacizumab treatment across the iTTP care pathway. Transitions between care units, prescription processes, pharmacy supply, and outpatient treatment may create treatment gaps; coordinated communication and secured drug availability are therefore essential.

To prevent exacerbations and potentially fatal complications related to unintended caplacizumab interruptions, continuous drug availability and reliable communication across transitions of care are essential. Medication administration errors may arise from workflow disruptions, communication failures, and supply problems [14,15,16,17,18,19,20,21,22]. Our cases illustrate these vulnerabilities: in both patients, at least one missed dose was related to drug unavailability. Clear coordination among physicians, nurses, pharmacists, and the involved care units, together with procedures that ensure uninterrupted caplacizumab supply, may reduce avoidable treatment gaps (Figure 2).

With regard to intended interruption or cessation of caplacizumab therapy, ADAMTS13 activity is the most reliable surrogate marker of underlying iTTP disease activity. Treatment decisions should therefore not rely solely on clinical improvement or normalization of platelet count and LDH. Persistently low ADAMTS13 activity indicates ongoing immune-mediated disease and continued vulnerability to recurrence when caplacizumab is withdrawn. Serial ADAMTS13 monitoring should consequently guide treatment duration, particularly after PEX has been discontinued [9,23]. In our two cases, the unintended interruptions occurred while ADAMTS13 activity remained severely reduced, supporting the concept that this early phase is particularly vulnerable.

In conclusion, we described two illustrative cases of iTTP with interruption of caplacizumab therapy. Both cases support our hypothesis that unintended treatment interruptions, particularly at low ADAMTS13 activity, contribute to suboptimal outcomes. Awareness of the importance of uninterrupted caplacizumab administration should be established among all healthcare professionals and disciplines involved throughout the treatment pathway, accompanied by measures to ensure continuous drug availability.

## Data Availability

The data presented in this study are available on request from the corresponding author due to patient privacy and confidentiality considerations.

## Abbreviations

CHD	Coronary heart disease
CT	computed tomography
cTTP	congenital TTP
ICU	intensive care unit
iTTP	immune-mediated TTP
LDH	lactate dehydrogenase
PEX	plasma exchange
TTP	thrombotic thrombocytopenic purpura
UL-VWF	ultralarge-von-Willebrand-factor
VWF	von Willebrand factor
